# Long-term results of cementless humeral head resurfacing for humeral head osteonecrosis – a monocentric longitudinal observational study

**DOI:** 10.1007/s00264-025-06622-0

**Published:** 2025-08-18

**Authors:** Matthias Bülhoff, Amina Gurda, Johannes Weishorn, David Spranz, Kevin Knappe, Patric Raiss, Raphael Trefzer

**Affiliations:** 1 Heidelberg University, Heidelberg, Germany; 2 Orthopädische Chirurgie München, Munich, Germany

**Keywords:** Osteonecrosis, Humeral head, Stemless, Resurfacing, Hemiarthroplasty, Glenoid erosion

## Abstract

**Purpose:**

Humeral head osteonecrosis (HHN) is a joint-destructive condition, for which cementless humeral head resurfacing (CHHR) offers a bone-preserving treatment option. The aim of this study was to report long-term outcomes and implant survival of CHHR in patients with HHN.

**Methods:**

Patients with humeral head osteonecrosis treated with cementless humeral head resurfacing (CHHR) between 2004 and 2007 were included. Implant survival was assessed according to Kaplan-Meier analysis. Clinical evaluation included Constant-Murley-Score (CMS), Simple Shoulder Test (SST), Subjective Shoulder Value (SSV) and patient centered outcomes regarding satisfaction and quality of life. Radiographs were evaluated for glenoid erosion, Walch glenoid types as well as signs of implant loosening. Statistical comparison was performed using students t-tests with a significance level set to *p* < 0.05.

**Results:**

Seventeen shoulders were retrospectively included in the implant survival analysis. Two patients underwent revision surgery. five patients died with the implant and were therefore censored. Cumulative survival rate was 100% after ten years and 93.3% after 15 years. Seven shoulders were available for clinical and radiological evaluation at a mean follow-up of 19 years (range 17–22 years). Age- and sex-adjusted CMS improved from preoperative to the latest follow-up (44.4% vs. 82.9%; *p* < 0.01). No glenoid erosion of higher degree (Sperling grade > 2) and no signs of implant loosening were observed. All patients had Walch type A glenoids preoperatively.

**Conclusion:**

In this small cohort with long-term follow-up of 17–22 years, CHHR showed promising durability and functional outcomes in carefully selected patients.

**Level of evidence:**

Level IV Case series with no comparison group.

## Introduction

Humeral head necrosis (HHN) is a rare condition which can affect younger patients presenting significant challenges to treating physicians, making accurate diagnosis and timely therapy critical for achieving favourable outcomes [1].

Conservative measures, which may be appropriate in the early stages [2], can be escalated to minimally invasive procedures such as arthroscopic debridement, retrograde drilling, or vascularized bone graft transplantation [3–6]. A more recent therapeutic option is collagen matrix implantation surgery [7]. In advanced stages or cases involving extensive necrosis, joint replacement surgery becomes particularly important.

Shoulder arthroplasty is considered the gold standard for managing end-stage shoulder degeneration, even in the long-term [8–10]. Although the results of shoulder arthroplasty for osteoarthritis (OA) are well studied, there is limited research on the long-term outcomes of shoulder arthroplasty in patients with HHN. Studies examining the outcomes of total shoulder arthroplasty in this population suggest that glenoid replacement is more effective in reducing pain than procedures without it [11, 12]. Mid-term data on hemiarthroplasty (HA) indicate good to excellent results, with high patient satisfaction [11, 13].

Cementless Humeral Head Resurfacing (CHHR) offers the advantage of preserving bone structure, which especially becomes relevant in younger patients with focal disease and intact glenoid cartilage. Data, including results from implant designers, demonstrate satisfactory outcomes across various pathologies [14, 15]. In cases of highly localized and well-demarcated necrosis, CHHR may be a suitable option [16]. Malperfusion of the humeral head has raised concerns regarding the fixation of cementless stemless implants, and long-term data on the risk of aseptic loosening due to microcirculatory disturbances in the etiology of HHN are scarce.

The objective of this study was to evaluate long-term clinical outcomes, radiographic findings, and implant survival following CHHR in patients with HHN.

## Methods

### Patients

Seventeen shoulders (15 patients) treated with CHHR for humeral head osteonecrosis who were recorded in a local monocentric database between March 2002 and October 2005 were included in this retrospective longitudinal study. The short- to mid-term results of this cohort were already published previously [17]. Patients who underwent revision surgery with implant removal or conversion to total shoulder arthroplasty with implantation of a glenoid component were excluded from long-term follow-up examination but were included in the survival analysis. Patients with unknown implant status were documented as “lost to follow-up”.

**Fig. 1:**
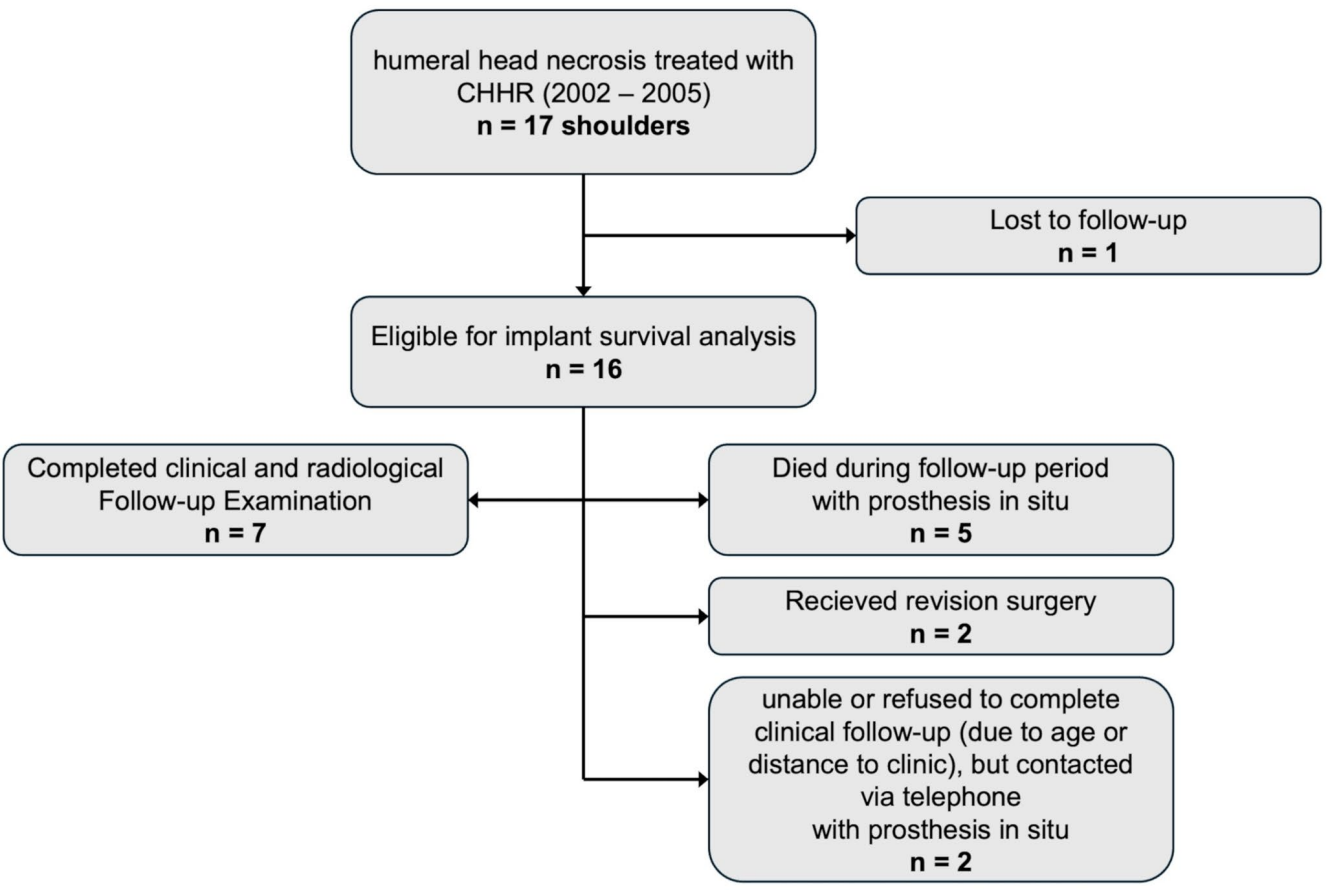
Study cohort flow diagram. CHHR = Cementless humeral head resurfacing

Seven shoulders completed the long-term follow-up examination including clinical and radiological evaluation at a mean follow up of 18.8 years. Five patients (5 shoulders) had died within the follow-up period with the prosthesis in situ, two patients (2 shoulders) were not able to come to the follow-up examination because of illness, age or long travel distance to the clinic. One patient was lost to follow-up as her prosthesis status was unknown at the time of the data collection. Two patients needed revision surgery: One patient that was initially treated for posttraumatic HHN was revised to Reverse Shoulder Arthroplasty (RSA) due to glenoid erosion and a massive cuff defect with decentered joint. The other patient needing revision was initially treated for posttraumatic HHN and received revision to RSA due to a traumatic periprosthetic proximal humerus fracture. The patient flow chart is depicted in Fig. 1.

All patients signed informed consent preoperatively upon entry in the database. Ethical approval was obtained from the local Institutional Review Board (S305-2007). The study was conducted in accordance with the Helsinki Declaration of 1975, as revised in 2013. Informed consent was obtained from all participating patients. No funding was obtained for this study.

### Surgical technique

All patients were operated in the beach-chair position via a deltopectoral approach with subscapularis tendon detachment and capsular release to expose the joint. Intraoperative findings confirmed the preoperative indication in all cases. Tenodesis of the long head of the biceps tendon with soft-tissue fixation in the bicipital groove was performed in each patient. The humeral head was reamed with hemispherical reamers of decreasing size for the Copeland Shoulder (Biomet Europe, Dordrecht, The Netherlands) and Epoca RH Cup (Argomedical, Cham, Switzerland) prostheses, which are the implant systems used in the present study. The implant choice between the two prostheses was based on availability.

Sites of osteonecrosis were addressed using cancellous bone grafting which was harvested during reaming, and cementless implants were impacted using a press-fit technique. Primary stability was manually assessed and confirmed after implantation. Subscapularis repair was achieved with tendon-to-tendon sutures.

Drains were removed on the first postoperative day. To protect the reconstructed subscapularis tendon, the arm was positioned in internal rotation on a shoulder abduction pillow for four weeks. Postoperatively, passive mobilization was initiated, with gradual increases to 60° of flexion and abduction and 0° of external rotation over six weeks, with patients encouraged to actively support these movements. Full range of motion was released at six weeks post-surgery.

### Clinical evaluation and patient reported outcome measures (PROMs)

Long-term clinical outcome was evaluated by one observer with Constant-Murley Score (CMS) including the respective subcategories: Pain, Activities of Daily Life, Mobility, strength [18]. CMS points were additionally calculated as age- and gender-adjusted CMS (aCMS) [19]. Isometric abduction strength was measured with the arm in 90° abduction using an Isobex dynamometer (Cursor AG, Bern, Switzerland).

Patient satisfaction was surveyed using a four answer based questionnaire (very satisfied, satisfied, undecided, unsatisfied). Patients self-reported their perception of shoulder mobility, activity level and function using the Simple Shoulder Test (SST) and Subjective Shoulder Value (SSV). Health related quality of life after surgery was assessed using the EQ-5D-3 L questionnaire [20].

### Radiological evaluation

Radiological assessment was performed using standard anteroposterior (true a. p.) and axial radiographs of the shoulder preoperatively, at the short- to mid-term and the long-term follow-up by one observer that was blinded to the clinical outcomes. Signs of loosening, defined as radiolucent lines with implant migration or tilt compared to previous radiographs, and glenoid erosion was evaluated. The extent of glenoid erosion was classified in four grades according to Sperling [21]. The wear patterns and glenoid deformity were descriptively characterized according to Favard classification [22]. Glenoid types according to Walch were determined preoperatively using the preoperative standard radiographs [23]. The joint reconstruction parameters of lateral glenohumeral offset (LGHO), defined as the distance from the lateral edge of GT to the base coracoid process, and humeral offset (HO), defined as the distance from the lateral edge of the greater tuberosity (GT) to the centre of rotation, were measured in preoperative and postoperative anteroposterior radiographs as previously described by Maier et al. [24]

### Statistical evaluation

SPSS Version 26.0 (IBM^®^, Armonk, New York, USA) was used for statistical analyses. Baseline demographic parameters were analyzed descriptively. Paired student’s t-test was used for comparative statistical analysis of preoperative, short-term and long-term follow-up parametric normally distributed data of CMS and radiological measurements. Cumulative implant survival was calculated using Kaplan-Meier analysis with revision for any reason as the endpoint. 95% confidence intervals (95% CIs) were calculated. Patients who died with the prosthesis in situ were censored. All analyses were conducted with the significance level set to *p* < 0.05.

## Results

### Patients

In the cohort of 17 shoulders (15 patients), six patients (9 shoulders) were diagnosed with metabolic HHN (Table 1). According to Cruess types, there was one shoulder classified as grade 2, seven shoulders as grade 3 und one shoulder as grade 4 in the metabolic group [25]. Five patients (7 shoulders) had chemotherapeutic treatment in their history (3 patients (4 shoulders) due to Hodgkin-lymphoma, one patient (1 shoulder) due to non-Hodgkin-lymphoma, one patient (2 shoulders) due to acute myeloid leukemia). One patient (2 shoulders) had high-dose corticoid treatment in the history due to hypophysis adenoma. Eight patients (8 shoulders) were diagnosed with posttraumatic avascular HHN after proximal humerus fracture; six of them received initial osteosynthesis and two were initially treated conservatively. Three shoulders were classified as Cruess type 3 and five as type 4 in the group with posttraumatic HNN.

**Fig. 2:**
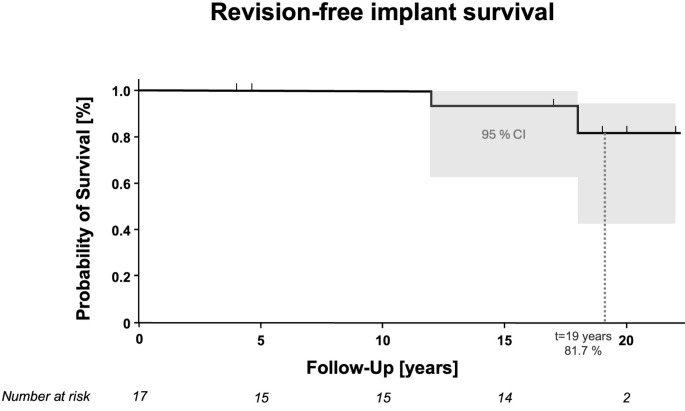
Kaplan-meier curve of estimated cumulative revision-free implant survival rate. I Indicates censoring

Cumulative revision-free implant survival was 100% at ten years (number at risk: 15), 93.3% (CI 61.3–99.0) at 15 years (number at risk: 14) and 81.7% (CI 42.0–95.4) at the mean follow-up of 19 years (number at risk: 4) (Fig. 2).

### Clinical evaluation and proms

The seven shoulders with complete clinical follow-up exhibited statistically significant improvement of CMS as well as aCMS and the CMS subcategories pain, activities of daily life, mobility and strength compared to preoperative (Table 2). No change in aCMS was found over the postoperative course compared to the short-/mid-term outcome (Table 2). ROM also showed a stable improvement from short to long term compared to preoperative for flexion, abduction and external rotation (Table 2).

four patients (5 shoulders) reported to be “very satisfied” with the operation and one patient (2 shoulders) reported to be “satisfied”. Relative SST averaged 83% (73–95%), SSV averaged 60% (30–90%). Health-related quality of life was evaluated using the EQ-5D-3 L (German value set, Greiner et al., 2005 [26]). The mean utility score was 0.92 (SD ± 0.04), mean VAS was 75% (SD ± 18).

### Radiological evaluation

**Fig. 3:**
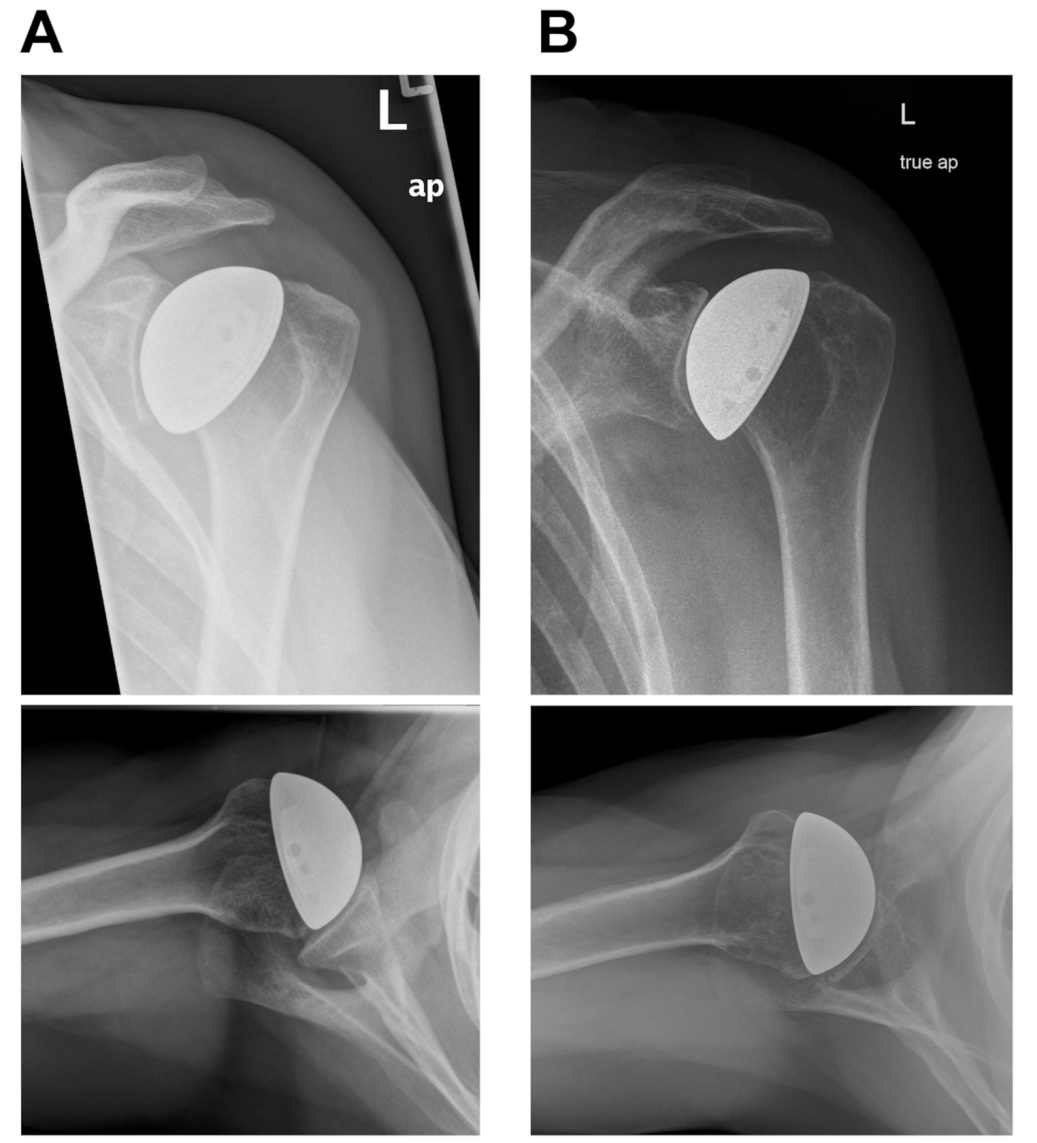
Radiological results of a postoperative course: (**A**) postoperative true a. p. (upper) and axial (lower) radiograph of a female patient operated at the age of 41 with diagnosed humeral head osteonecrosis Cruess stage 3 due to Hodgkin lymphoma treated with chemotherapy. (**B**) Follow-up radiograph true a. p. (upper) and axial (lower) at 17 years postoperatively showing mild glenoid erosion and a centered joint

All 17 shoulders had a centered glenohumeral joint preoperatively according to the Walch classification (13 shoulders Type A1, four shoulders Type A2). Amongst the seven shoulders with complete clinical and radiological follow-up, six showed mild glenoid erosion and one showed none according to Sperling (Fig. 3). The quality of glenoid erosion was rated as E1 for four shoulders and E0 for threeshoulders. No signs of loosening were observed. Offset reconstruction parameters were not changed significantly from preoperative to postoperative, however a reduction in LGHO in postoperative course was observed (Table 3).

## Discussion

To the best of our knowledge, this is the first case series to report long-term results at 19 years in patients treated with CHHR for HHN. The data show promising durability and functional outcomes in carefully selected patients at 19 years of follow-up. In addition, no implant-specific complications or radiographic sings of loosening have been observed with the stemless implant designs.

As outlined above, this study completes the long-term follow-up to the prior investigation by Raiss et al. [17]. At three years, the same cohort demonstrated an aCMS of 75%, with fifteen patients reporting being very satisfied or satisfied at the final follow-up. No evidence of implant loosening was observed, which is conistent with our findings [17].

Data evaluating patients with humeral head necrosis (HHN) undergoing shoulder arthroplasty are scarce in the current literature. A large retrospective Medicare database study by Parel et al. reported satisfactory outcomes in patients with avascular HHN undergoing total shoulder arthroplasty, both anatomic and reverse. Furthermore, good implant survival rates were observed at ten years postoperatively [27]. These results are also supported by a multinational study by McLaughlin et al. [28].

The development group of humeral head resurfacing with Levy et al. presented data on 285 implants with a mean follow-up of 6.8 years, covering various diagnoses and treatments, including hemiarthroplasty and total shoulder arthroplasty. In their cohort, aCMS scores reached 93.7% in patients who underwent additional glenoid replacement, while patients who underwent hemi humeral head resurfacing had an aCMS of 73.5%. Radiological loosening was observed in three implants at the final follow-up [14]. The same group presented results from young patients with OA who were treated with an HHR and had a minimum follow-up of ten years (average 12.5 years) [29]. In a subgroup, 16 patients with humeral head necrosis were treated, achieving a mean Constant Score of 75 points at the final follow-up. One patient required revision surgery due to a fracture, and another after 16 years due to secondary glenoid erosion. Flexion was almost 150 degrees at the final follow-up. Revision-free survival at 14 years of Follow-Up was 91%. These findings are comparable to our data. However, the presented study shows a longer follow-up for a specific selected patient population. Furthermore, a possible bias due to data from the development group must be noted [29].

Short-stem prostheses or stemless implants are increasingly being used nowadays, allowing for anatomical reconstruction of the proximal humerus. A study by Beck et al. presents long-term data from patients who were treated the stemless TESS prosthesis with an average follow-up of almost eight years, and a mean Constant Score of 68.8 points [30]. This cohort included three patients with humeral head necrosis. Long-term data are pending. The data agree with our findings, however follow-up in our study is longer and the number of specific patients is larger.

Another study evaluated the results of humeral head resurfacing hemiarthroplasty in 56 shoulders, with various diagnoses and a mean follow-up of almost three years. The aCMS improved to 54% at final follow-up, but four patients required further surgery: three arthroscopic and one due to implant loosening [15]. These results are difficult to compare, as they include different diagnoses and Levy’s study also included total shoulder arthroplasty with glenoid replacement implants.

Studies specifically examining outcomes based on the aetiology of the disease are limited. However, evidence suggests that patients with posttraumatic HHN tend to have worse outcomes than to those with atraumatic HHN [17, 31]. In particular, younger patients appear to be at a higher risk of requiring reoperation [12]. In our cohort, the small sample size and limited statistical power precluded further subgroup analyses.

A study by Mansat et al. demonstrated superior outcomes in patients with atraumatic HHN undergoing total shoulder arthroplasty compared to those treated with shoulder hemiarthroplasty, with a mean follow-up of seven years [11]. Although the study group was small (*n* = 19), the reported mean aCMS of 78% was comparable to our findings. Conversely, Schoch et al. observed a higher risk of implant revision and higher complication rates in patients who received TSA compared to HA for atraumatic HHN, suggesting that HA might be beneficial in atraumatic HNN patients with intact glenoid status [32]. This is supported by the low rates of glenoid erosion and low revision rates in our study cohort.

The CHHR used in this study showed no implant-specific complication on the humeral side. This is supported by previous studies showing good osseointegration of stemless humeral implants [33], and no humeral side loosening in young active patients [34]. The revision-free implant survival rate found in this study with two shoulders receiving revision is rather underestimated, as more shoulders (5) were censored because of death within the follow-up period. Due to the small cohort size, a competing risk analyses was not performed.

Radiological evaluation of the offset parameters showed adequate postoperative reconstruction of HO and LGHO. While HO showed a stable course over the long-term follow-up, LGHO decreased slightly from postoperative to long-term follow-up. This might be due to loss of glenoidal offset caused by erosion of the glenoid cartilage and subchondral bone. The mild glenoid erosion observed in the presented study showed no clinical relevance in this specific cohort. High rates of glenoid erosion and low satisfaction rates were reported for patients treated with stemmed hemiarthroplasty [35]. However, these patients were treated for glenohumeral OA and dissatisfaction was specifically observed in patients with eccentric glenoid wear, highlighting that an adequate reconstruction of the parameters mentioned above and a concentric glenoid conformation might be beneficial for a good clinical outcome in the long-term.

The main strengths of this designer-independent study are the long follow-up, with only one patient lost to follow-up, and the homogeneous and carefully seleceted patient cohort with the rare indication of HHN treated with CHRR.

The small number of patients limits the statistical power for further analyses of differences between subgroups or associations between radiological and clinical findings. Furthermore, only seven shoulders completed long-term follow-up, which is due to the high but reasonable mortality over the years and the inability of two patients to attend the follow-up visit. This limited cohort reduces the generalizability and statistical robustness of the results. The absence of a control group and the relatively wide confidence intervals in the survivorship analysis further constrain interpretability. Consequently, effect sizes should be interpreted with caution and not overestimated.

Nevertheless, implant status could be determined in 16 out of 17 shoulders, providing valuable data for successful treatment with stemless HHR including appropriate implant selection. Despite its limitations, this study contributes uniquely to the existing literature by offering rare long-term data on this specific treatment approach in selected patients.

**Table 1:** Patient demographic data

	Mean (range)	*N* (%)
**total**		17 (100%)
**Age at surgery [years]**	40 (35–82)	
**Female sex**		9 (53%)
**Follow-up total [years]**	16.25 (0.25–22)	17 (100%)
Follow-up clinical examination [years] *	18.8 (17.1–22)	7 (41%)
**Diagnosis: metabolic / posttraumatic HHN**		9 (53%) / 8 (47%)
**Cruess grade 2**		1 (6%)
**Cruess grade 3**		10 (59%)
**Cruess grade 4**		6 (35%)
**Dominant hand side affected**		8 (47%)

HHN = Humeral head necrosis. Data refer to the total cohort of all 17 shoulders except for the follow-up of the subgroup at the final clinical assessment (*)

**Table 2:** Clinical outcomes

	preoperative		short-term Follow-up^†^		long-term Follow-up		Preop. vs. long-term FU
	mean	SD	mean	SD	mean	SD	*P*-value
**CMS [points]**	39.6	8.2	67.4	15.6	65.7	16.3	* 0.002
**aCMS [%]**	44.4	11.7	77.6	19.2	82.9	24.1	* 0.002
**CMS pain [points]**	5.0	3.7	13.6	3.5	12.4	3.5	* <0.001
**CMS activity [points]**	11.6	3.9	18.6	1.8	14.7	4.3	*0.054*
**CMS mobility [points]**	19.1	5.6	31.1	8.5	31.1	7.5	* 0.002
**CMS strength [points]**	3.9	4.7	5.1	3.5	7.4	5.5	*0.301*
**Flexion [°]**	107.1	29.6	147.9	26.7	141.4	42.1	*0.064*
**Abduction [°]**	75.7	28.7	137.1	32.8	125.7	37.8	* 0.019
**External rotation [°]**	-1.4	17.3	24.3	11.8	36.0	16.2	* 0.007

CMS = Constant-Murley-Score, aCMS = age- and sex-adjusted CMS, sst = simple shoulder Test, ssv = subjective shoulder value. † short-term follow-up refers to the previous report of clinical outcomes whole study cohort (*n* = 17) at three years postoperatively by Raiss et al. [17]

**Table 3:** Radiological outcome of joint offset reconstruction

	preoperative		postoperative		long-term Follow-up	
	mean	SD	mean	SD	mean	SD
**HO [mm]**	30.8	2.7	32.7	3.7	30.3	6.2
	pre- vs. postop.: *p* = 0.36			postop. vs. long-term FU: *p* = 0.31		
**LGHO [mm]**	71.8	6.0	73.7	10.7	61.7	7.8
	pre- vs. postop.: *p* = 0.66			postop. vs. long-term FU: *p* = 0.03		

HO = Humeral offset, lgho = lateral gleno-humeral offset

## Conclusion

CHHR provides promising durability and functional outcomes with and high patient satisfaction at 19 years in carefully selected patients with metabolic or posttraumatic HHN. Furthermore, implant survival with this treatment option is high with no implant-specific complications observed in this study cohort. With adequate offset reconstruction and intact preoperative centered glenoid status, glenoidal degeneration is low in these patients.

## Data Availability

No datasets were generated or analysed during the current study.
